# Quadricuspid Aortic Valve: A Rare Case of Endocarditis Suspicion and Management

**DOI:** 10.7759/cureus.64419

**Published:** 2024-07-12

**Authors:** Seyi A Olaniyi, Anne Saidu, Seun Arowolo, Alen Sam, Chinyere K Omeh, Sofia Ali, Misbah Kamal Khan

**Affiliations:** 1 Medicine and Surgery, Obafemi Awolowo University, Ile Ife, NGA; 2 Medicine, V.N. Karazin Kharkiv National University, Kharkiv, UKR; 3 Internal Medicine, Obafemi Awolowo University, Ile Ife, NGA; 4 Internal Medicine, Government Medical College, Kozhikode, Kozhikode, IND; 5 Internal Medicine, Nigerian Airforce Hospital, Makurdi, NGA; 6 Medicine, Peninsula Medical School, Plymouth, GBR; 7 Internal Medicine, Peoples University of Medical & Health Sciences, Nawabshah, PAK

**Keywords:** congenital cardiac anomaly, aortic valve tricuspidization, aortic endocarditis, aortic regurgitation, quadricuspid aortic valve -

## Abstract

Quadricuspid aortic valve (QAV), a rare congenital cardiac anomaly, often presents with aortic regurgitation and can lead to significant cardiovascular complications. This case report describes a 55-year-old male with a history of subarachnoid hemorrhage who was incidentally found to have QAV with possible endocarditis. Transesophageal echocardiography revealed thickened leaflet tips on all four cusps and a mass on one leaflet, raising suspicion of endocarditis despite the absence of vegetation. The patient was treated with intravenous antibiotics for Gram-positive bacteremia, and follow-up imaging confirmed the QAV anomaly with moderate aortic regurgitation. This case highlights the challenges in diagnosing QAV, particularly in asymptomatic individuals, and underscores the need for comprehensive investigation, especially in those with a history of vascular events. It also emphasizes the importance of further research to clarify the long-term risks and optimal management strategies for individuals with QAV, including the potential for infective endocarditis.

## Introduction

Less than 1% of people have a quadricuspid aortic valve (QAV), an uncommon congenital abnormality [1]. The rarity of the QAV, a rare congenital cardiac disease, defines it. Aortic regurgitation (AR) is often the main functional problem linked to QAV. Depending on the degree of aortic valve failure and any other cardiac conditions, persons with QAV present with a variety of clinical symptoms and presentations. Notably, many QAV patients frequently experience severe valvular stenosis and/or regurgitation, necessitating surgical intervention frequently during their fifth and sixth decade of life. The QAV's leaflets have the potential to thicken and fibrose with time, which might lead to inappropriate leaflet coaptation and the emergence of AR [2]. Aortic stenosis, a narrowing of the aortic valve opening, is also occasionally possible although less often.

Diagnostic imaging methods have significantly improved in recent decades, notably in the area of ultrasound. QAV has been more widely known thanks to these developments, which have also made it possible to better understand how to diagnose it, how it progresses clinically, and how to manage it. Typically, echocardiography is used to diagnose QAV, while other imaging techniques can be required if the echocardiographic pictures are of low quality [3]. Hemodynamic effects, such as a dilated left ventricular chamber or a decline in left ventricular systolic performance, may occur in individuals with chronic AR [4]. How to treat individuals with a QAV is a topic of continuous debate and uncertainty. These discussions center on deciding whether surgery is necessary, choosing the best surgical technique, and choosing whether to use antibiotics to prevent infective endocarditis.

## Case presentation

A 55-year-old male with a past medical history of hypertension, psoriatic arthritis, rheumatoid arthritis, and bipolar 1 disorder now presented with a one-week history of dyspnea on exertion. The patient had a prior history of aneurysmal subarachnoid hemorrhage on March 21, 2022, which was clotted and stabilized. He has a past history of smoking, occasional alcoholic consumption, and a history of substance abuse, which includes methamphetamine. His prior surgical history includes a gastric bypass surgery, a knee surgery, a hip surgery, and a shoulder surgery. He has a family history of deep vein thrombosis and diabetes in his mother, and abdominal aortic aneurysm and hypertension in his father.

**Figure 1:**
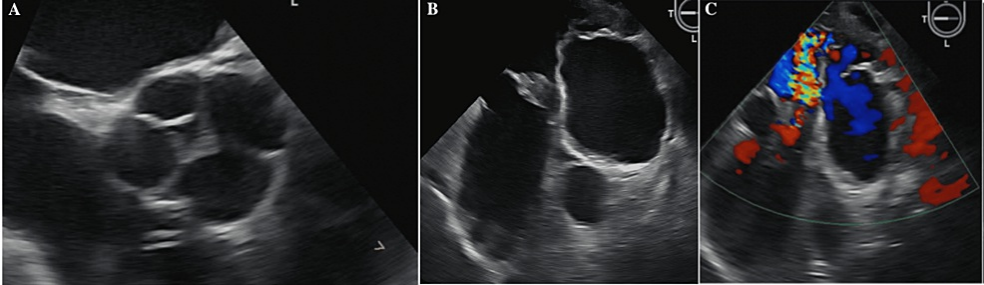
The transesophageal echocardiogram (TEE) revealed a unique abnormality of the aortic valve. The mid-esophageal aortic valve short-axis view (A) demonstrates a quadricuspid aortic valve (QAV). These leaflets also exhibit thickening at their tips, and a concerning 1.3 x 1 cm mass is attached to one of them (visible in both A and potentially B). Furthermore, the long axis view of the left ventricle (C) shows mild to moderate aortic regurgitation.

On clinical examination, the patient was well-built with normal pulmonary, cardiovascular, and neurological findings. A transesophageal echocardiogram revealed that he has a QAV with all the leaflet tips thickened. It also showed a 1.3 x 1 cm mass present on one leaflet that did not seem to be independently mobile as shown in Figures 1A-1C. Additionally, mild-to-moderate AR was present, which led to the possibility of endocarditis despite the lack of vegetative appearance of the mass.

During the period of hospitalization, the patient was diagnosed with Gram-positive bacteremia and was managed with intravenous antibiotics through a PICC line. The follow-up echocardiogram showed quadricuspid valve anomaly with thickening of the leaflets and the mass attached along with moderate AR as shown in Figure 1. No intracardiac thrombus was seen. The plan was to complete the rest of the antibiotic prescription, order a cardiac MRI to further investigate the aortic valve abnormality and regurgitation, and possibly refer to cardiothoracic surgery based on the results of the investigation.

## Discussion

The reported frequency of QAV, a rare heart abnormality, differs depending on the method of diagnosis. The prevalence suggested by cardiac ultrasonography studies, which ranges from 0.013% to 0.043% but is still the closest estimate to the actual prevalence, is probably underestimated [2]. Contrary to popular belief, the pulmonic valve and truncus arteriosus both exhibit QAV more frequently than the aortic valve. Balmington's description of QAV in 1862 is the earliest known record of the condition [5]. Studies reveal that aberrant arteriopulmonary septation fusion and abnormal mesenchymal proliferation may both offer viable reasons for the formation of QAV when looking at its embryologic beginnings [6].

Although it can coexist with other sporadic cardiac diseases, a QAV is often identified as an independent abnormality. When compared to trileaflet aortic valves, there is an anticipated variation in the location of the coronary artery openings in relation to the aortic sinus of Valsalva in QAV. Although it is frequently seen in trileaflet aortic valves, the physical location of coronary artery origins in relation to the aortic root is typically identical. Coronary ostia displacement has been reported, including cases with a shared etiology; however, these instances seem to be unusual. However, it is useful to precisely define the location of the coronary artery openings, especially when considering surgical procedures [7,8]. The aortic valve is best visible in short-axis cardiac ultrasound scans because it provides the most accurate diagnosis and delineation. Transesophageal echocardiography (TEE) examination can provide a more precise degree of detail when the transthoracic echocardiography (TTE) results are not totally evident. Important details such as the degree of AR, the size and functionality of the left ventricle, the location of the coronary ostia, the presence of any associated cardiac abnormalities, aortic stenosis (AS), and the state of the ascending aorta, can all be revealed by ultrasound imaging [9].

The presence of severe AR, severe aortic stenosis, or a defective QAV with concomitant problems such as a blocked left coronary ostium is often the main justifications for surgical intervention. Many of these patients, notably those with persistent, severe AR, need to have their aortic valves replaced [10]. The surgical procedure known as “aortic valve tricuspidization,” which entails the removal of the auxiliary cusp and the merging of the remaining three cusps, is the most frequently used to treat QAV. There have also been reports of the use of a procedure known as “bicuspidization,” in which the commissural closures of the valve are stitched together to produce a bicuspid aortic valve [11].

Despite some findings suggesting otherwise, the possible risk of infective endocarditis in people with QAVs is still unknown. According to certain case studies, valves with smaller accessory cusps may be at a higher risk because of unequal stress distribution and aberrant leaflet coaptation [12]. Infective endocarditis prophylaxis is not currently advised for these individuals [13].

## Conclusions

This case report underscores the elusive nature of QAV, a rare congenital anomaly often discovered incidentally during unrelated investigations. The patient's presentation with neurological symptoms led to the unexpected diagnosis of QAV, potentially complicated by endocarditis. This case serves as a reminder of the importance of maintaining a broad differential diagnosis and considering rare conditions in patients with atypical presentations. The diagnostic workup of QAV often involves a combination of imaging modalities, with echocardiography playing a pivotal role. However, additional imaging, such as cardiac MRI, may be necessary to delineate the valve anatomy and guide treatment decisions. While surgical intervention is often indicated for symptomatic QAV, the optimal management approach remains a subject of ongoing debate and research. Further studies are needed to elucidate the long-term risks, including the potential for infective endocarditis, and to refine evidence-based treatment guidelines for individuals with QAV. This case report contributes to the growing body of literature on this rare condition and highlights the need for continued research and clinical vigilance to improve patient outcomes.
